# Predicting scalar coupling constants by graph angle-attention neural network

**DOI:** 10.1038/s41598-021-97146-1

**Published:** 2021-09-21

**Authors:** Jia Fang, Linyuan Hu, Jianfeng Dong, Haowei Li, Hui Wang, Huafen Zhao, Yao Zhang, Min Liu

**Affiliations:** 1 School of Physics and Electronics, Central South University, Changsha, 410083 China; 2 College of Computer Science and Technology, Zhejiang University, Hangzhou, 310007 China; 3 School of Economics and Management, Beijing Jiaotong University, Beijing, 100044 China; 4 College of Computer and Information Engineering, Zhejiang Gongshang University, Hangzhou, 310018 China

**Keywords:** Computer science, Atomic and molecular physics, Statistical methods, Cheminformatics

## Abstract

Scalar coupling constant (SCC), directly measured by nuclear magnetic resonance (NMR) spectroscopy, is a key parameter for molecular structure analysis, and widely used to predict unknown molecular structure. Restricted by the high cost of NMR experiments, it is impossible to measure the SCC of unknown molecules on a large scale. Using density functional theory (DFT) to theoretically calculate the SCC of molecules is incredibly challenging, due to the cost of substantial computational time and space. Graph neural networks (GNN) of artificial intelligence (AI) have great potential in constructing molecul ar-like topology models, which endows them the ability to rapidly predict SCC through data-driven machine learning methods, and avoiding time-consuming quantum chemical calculations. With a priori knowledge of angles, we propose a graph angle-attention neural network (GAANN) model to predict SCC by means of some easily accessible related information. GAANN, with a multilayer message-passing network and a self-attention mechanism, can accurately simulate the molecular-like topological structure and predict molecular properties. Our simulations show that the prediction accuracy by GAANN, with the log(MAE) = −2.52, is close to that by DFT calculations. Different from conventional AI methods, GAANN combining the AI method with quantum chemistry theory (Karplus equation) has a strong physicochemical interpretability about angles. From an AI perspective, we find that bond angle has the highest correlation with the SCC among all angle features (dihedral angle, bond angle, geometric angles) about multiple coupling types in the small molecule datasets.

## Introduction

Scalar coupling constant (SCC), mediated by the bond electrons in a molecule^1,2^, describes the interaction between two magnetic nuclei in the nuclear magnetic resonance (NMR) spectroscopy^3–6^. The value of SCC varies with the type of coupled atoms and the number of bonds between the coupled atoms. In the molecular field, accurately measuring SCC contributes to elucidations of molecular structures. Generally, ^N^J_AB_ is used to denote the SCC between the atom A and atom B. The superscript N denotes the number of chemical bonds between the two coupled atoms^4,7,8^.

**Figure 1:**
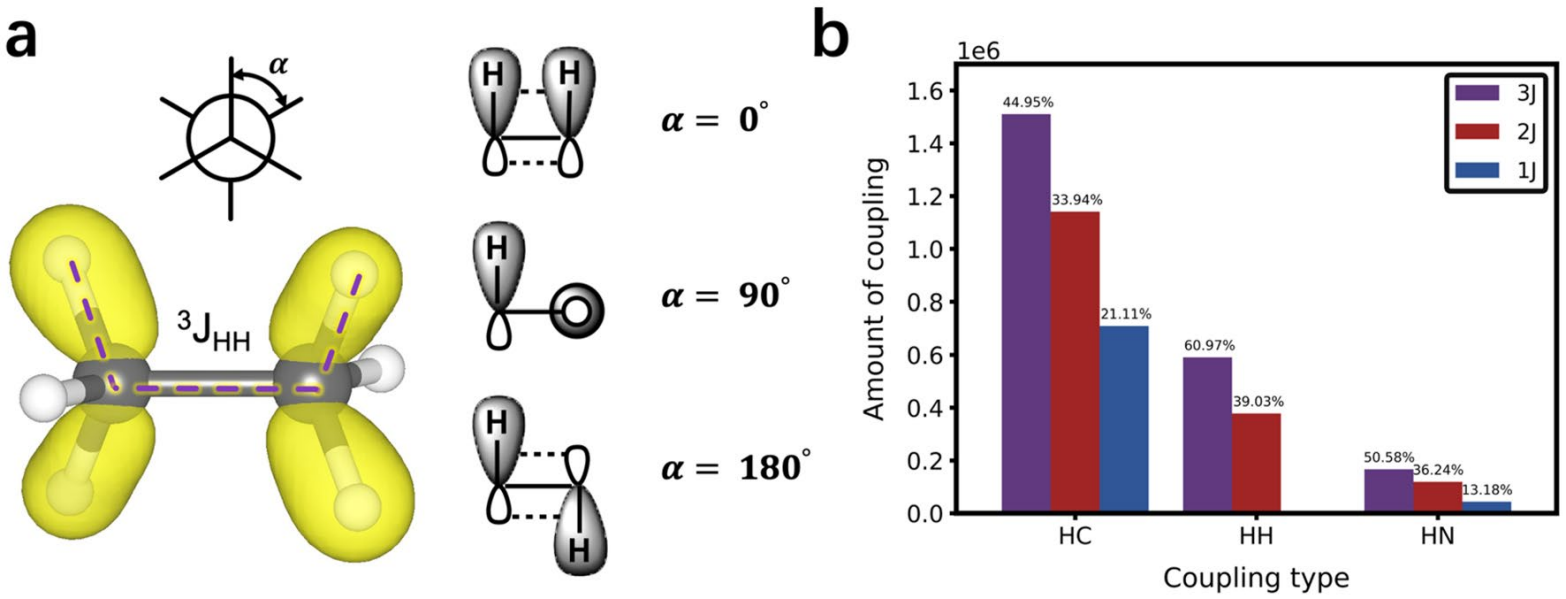
The relationship between the ^3^J_HH_ and the hybrid orbital angle (α), and statistics of coupling type. (**a**) The relationship between the SCCs and the angles of the hybrid orbitals. α is the angle at which the two hybridized orbitals form. The red dotted line represents that the scalar coupled magnetic interaction between two coupled hydrogen atoms is mediated by chemical bonds in the ethane molecule. The yellow irregular spindles represent the electron density distribution near the Fermi energy level of ethane. (**b**) Statistical description of SCCs in the dataset. 1 J, 2 J and 3 J couplings correspond to the blue, red and purple cylindricities respectively. Although 3 J accounts for a large proportion, the sum of 1 J and 2 J also accounts for nearly half of the proportion in each coupling type.

The well-known Karplus equation establishes the quantitative relationship between 3 J coupling constant and the dihedral angle formed by coupled atomic planes^9–11^, whereas it is not applicable to 1 J and 2 J coupling constants. Since SCCs are affected by many factors, it is difficult to unify and conclude a universal rule for all coupling types. The underlying rationale of Karplus equation can be explained by the hybridized orbitals angle as Fig. 1a shown. The highest SCC occur with a hybridized orbitals angle of either 0° or 180°, and the lowest SCC occur at 90° with minimal orbital overlap^12,13^. According to the statistics of coupling types in the predicting molecular properties dataset^14^, 3 J coupling accounts for a large proportion, whereas the sum of 1 J and 2 J also accounts for nearly half of the proportion as shown in Fig. 1b. Although the Karplus equation only applies to 3 J coupling constants, it kindles an enlightenment that the relevant angles play a crucial role of influencing all types of SCCs (1 J, 2 J, 3 J and etc.). In general, molecular structures affect the SCC via geometric configuration and electronic structure. The former corresponds to bond length and bond angle, and the latter includes electronegativity of substituents and hybridization of orbitals^9,12,15^. Both the geometry configuration and the hybrid orbitals in the electronic structure indicate that the SCCs have close correlations with relevant angles (including bond angle, hybridization orbital angle and etc.). All in all, we obtain that the relevant angles between two coupled nuclei are closely related to the SCCs.

Experimentally, a large number of methods has been developed for the determination of SCCs based on the NMR spectroscopy^4^. Due to the high cost, experimental methods are generally not used for large-scale molecular structure analysis. Theoretically, the SCCs are calculated usually by using DFT methods. However, it remains a challenging task to efficiently determine the accurate values of SCCs especially for the system that consist of a large number of molecules with complex structures^2,16–18^.

Thanks to the boom of artificial intelligence (AI)^19–22^, AI models can predict SCCs on a large scale with some easily accessible relevant molecular information, avoiding time-consuming theoretical calculation methods^19,23–25^. One of the early attempts is the Associative Neural Networks model. This model integrated Multilayer Perception and K-Nearest Neighboring to predict various SCCs^26^. However, this method has a limitation in determining complex molecular structures and the reason is that two-dimension vector input of traditional machine learning cannot represent three-dimensional molecular structure well. After, Gerrard et al. applied Kernel Ridge Regression to predict ^1^J_CH_ within several seconds and achieve an MAE (2.01 Hz) without substantial deviations^8^. However, their work explores only one coupling type ^1^J_CH_, which is not applicable enough. Following their work, the Kaggle competition has collected a dataset^14^ including the most applicable coupling types (1 J, 2 J and 3 J) and organized global participants to predict SCC using AI methods. In the competition, numerous advanced and eye-catching deep learning and graph neural network (GNN) methods emerged. In order to predict the SCCs more accurately, Jaechang Lim et al. used a variant of message passing neural network (MPNN) and Andres Torrubia et al. constructed their model based on the standard Transformer architecture^14,27^. Although MPNN has a more flexible message passing mechanism than Transformer to simulate the unique structure of various molecules. It is incredibly challenging for MPNN to deal with complex macromolecules. In order to simulate the molecular bonding topology, Guillaume Huard et al. modified the MatErials Graph Network in predicting the SCCs^14^. The most striking, the winning team Jonathan Mailoa et al. took advantage of MPNN and Transformer, and reached the state-of-art by ensemble learning techniques^14^. Although their method achieves the highest accuracy, they are too complex to be applied in practice due to limited computational resources.

To sum up, GNNs has been widely adopted and proved to have strong prediction ability to predict SCCs among plenty of deep learning techniques. The reason is that GNNs can use the graph structure as input to simulate the topologies of various molecules^28–31^. Self-attention^27^, essentially a fully connected graph network, is skilled in dealing with complex macromolecular structures. These works provide a necessary enlightenment that combining the GNNs with self-attention mechanism can learn the molecular structure information fully, and avoids disadvantages of traditional GNNs in complex macromolecules^14^. Besides, though these methods achieve satisfactory accuracy, they lack sufficient interpretability^32,33^ of physical–chemical theories to be widely accepted by chemists. Exploring model interpretability contributes to better combing AI methods with physicochemical theories, and even discovering some potentially scientific laws^21,34^.

To include both the prediction accuracy and interpretability into the model, we propose a novel graph angle-attention neural network (GAANN) to predict SCCs. GAANN achieves a high prediction accuracy log(MAE) = −2.52,which is close to that by DFT calculations. The source code of our proposed GAANN model is available at https://github.com/FangJia0901/Bond-Angle-for-SCCs. Moreover, the results of our experiment demonstrate that bond angle has the highest correlation with the SCC among all angle features from an AI perspective.

## Results

### Overview of graph angle-attention neural network

**Figure 2:**
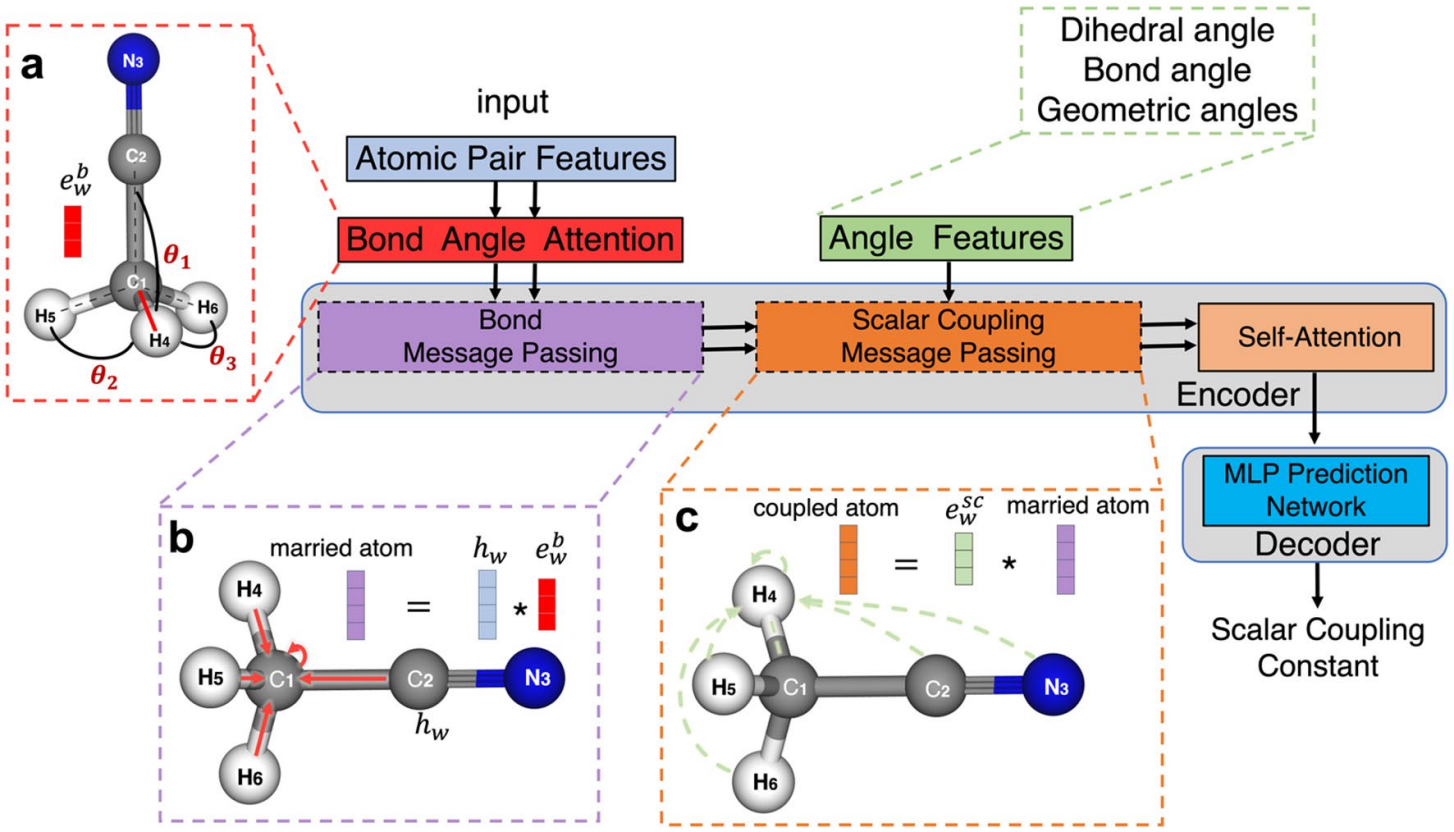
Overview of graph angle-attention neural network (GAANN). (**a**) (left) Bond Angle Attention. The red box corresponds to the bond angle attention in the encoder flow chart. (**b**) (center) Bond Message Passing. The purple box corresponds to the bond message passing layer. The neighbor's bonds $e_{w}^{b}$ depicted in the red feature vector are aggregated to update the centered atom C_1_ into the ‘married’ atom. (**c**) (right) Scalar Coupling Message Passing. The orange box corresponds to the scalar coupling message passing layer. The neighbor's scalar coupling edges $e_{w}^{sc}$ depicted in the green feature vector are aggregated to update the centered atom H_4_ into the ‘coupled’ atom. Next, the self-attention layer is used to extract the features of the ‘coupled’ atom in the yellow box. Finally, the purple box corresponding to the MLP is used to decode and predict the SCC.

To predict the value of SCCs, we propose a GAANN model which is a variant of the graph attention neural network^14,35^. The model architecture of GAANN is presented in Fig. 2. The whole framework of GAANN can be divided into the encoder part and the decoder part. The encoder part includes two-layer message passing neural networks and self-attention neural network. To highlight the importance role of relevant angles, we design bond angle attention in the first bond message passing layer and angle features in the second scalar coupling message passing layer.

As shown in Fig. 2b, we take acetonitrile as an example. In the bond message passing layer, each bond is aggregated to update the centered atom (C_1_) into the ‘married’ atom. Each bond, incorporated bond angle attention, is represented by the red feature vector $e_{w}^{b}$. In the Fig. 2a, the bond angle attention, inspired by the Karplus equation, can be regarded as a kind of prior physicochemical knowledge to vividly simulate the structure of bond angles in molecules^36^. Similarly, as shown in Fig. 2c, the structure of scalar coupling message passing is designed to simulate inter-atomic coupled interactions in molecules. The ‘coupled’ atom means the atom feature vector has combined with the coupled atoms’ status. The feature vector of ‘coupled’ atomic contains the information of coupled magnetic interactions between coupled atoms. In the scalar coupling message passing layer, angle features include dihedral angle, bond angle, geometric angles. After the two messages passing neural network, a self-attention is applied to the ‘coupled’ atom. Self-attention^27^, essentially a fully connected graph network, is the encoder's main framework to effectively extract the molecular features. Before decoding, we concatenate two coupled atoms features and the molecular features, then passed them through the multi-layer perception (MLP) to decode SCC.

### Prediction performance of GAANN

**Figure 3:**
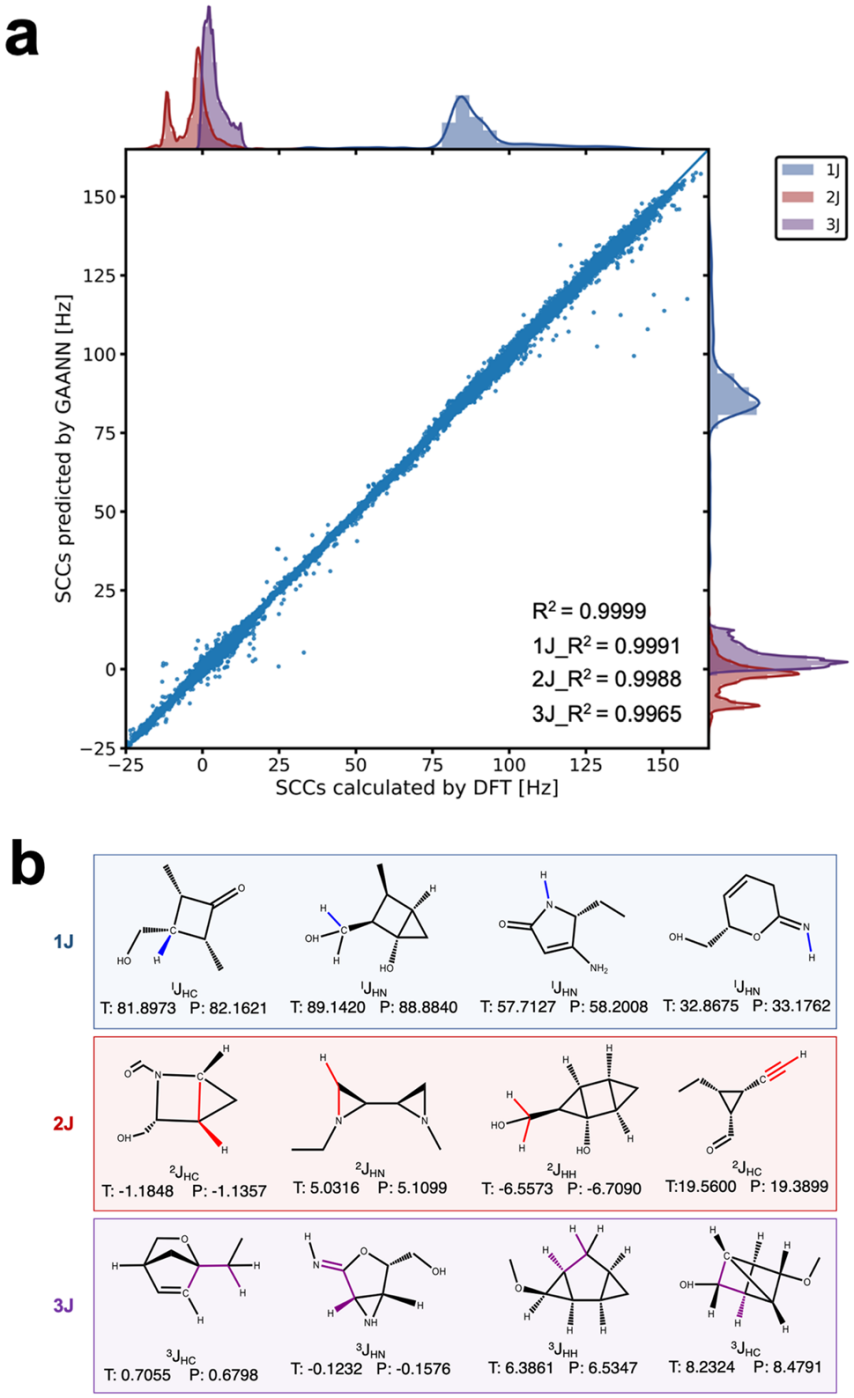
Evaluation of predicting performance of GAANN. (**a**) Plot of GAANN prediction versus DFT calculation. On the top and right of the figure, there are the kernel density curves of DFT goal values and prediction values respectively. Most of the scatter points are concentrated near the diagonal. (**b**) Twelve instances of DFT calculation (T) versus GAANN prediction (P). GAANN achieves satisfactory accuracy on three coupling types generally, and the margin of error is usually in the tenth place.

We demonstrate the prediction performance of GAANN on the whole in Fig. 3a. The abscissa is the goal SCCs computed by DFT, and the ordinate is the scalar coupling values predicted by GAANN. On the top and right of the Fig. 3a, there are the kernel density curves of DFT values and prediction values respectively. The blue kernel density curve shows that 1 J coupling mainly concentrates between 75 and 100 Hz. The red curve and purple curve show that 2 J and 3 J coupling concentrate between −20 Hz and 20 Hz. On the whole, the distribution of SCCs is a long-tail distribution. Therefore, log(MAE) is selected as evaluation criteria of prediction performance to avoid the sensitivity of outliers caused by the long-tail data^14^. The prediction accuracy of GAANN is −2.52 under the evaluation criterion of log (MAE).

In order to better show the performance of GAANN, we draw a scatter diagram between the predicted value and the target value. When the predicted value is close to the target value, the point will fall near the diagonal line. It can be seen from the Fig. 3a that most of the points are concentrated near the diagonal, and the scattered points are concentrated in the interval of [75 Hz, 100 Hz] and [−20 Hz, 20 Hz]. Generally, we use the determination coefficient *R*^2^ to evaluate the fitting degree between the predicted values and the goal values^37^. The value of *R*^2^ (0.999) indicates that the distribution of predicted value by GAANN is in good agreement with the distribution of the goal value by DFT calculation.

To demonstrate predictive performance of GAANN in detail, we randomly enumerated twelve examples of three coupling types (1 J, 2 J, and 3 J). Each coupling type includes J_CH_, J_NH,_ and J_HH_. As is exhibited in Fig. 3b, GAANN achieves satisfactory accuracy on three coupling types generally, and the margin of error is usually in the tenth place. Furthermore, the example shows that the larger the SCC, the smaller the prediction deviation. Although there is still a gap between the accuracy of GAANN and that of DFT calculation. Generally excessive pursuit of accuracy of SCCs is not required in preliminary analysis and screening of vast of molecular structures in practice.

### The embedding of GAANN

**Figure 4:**
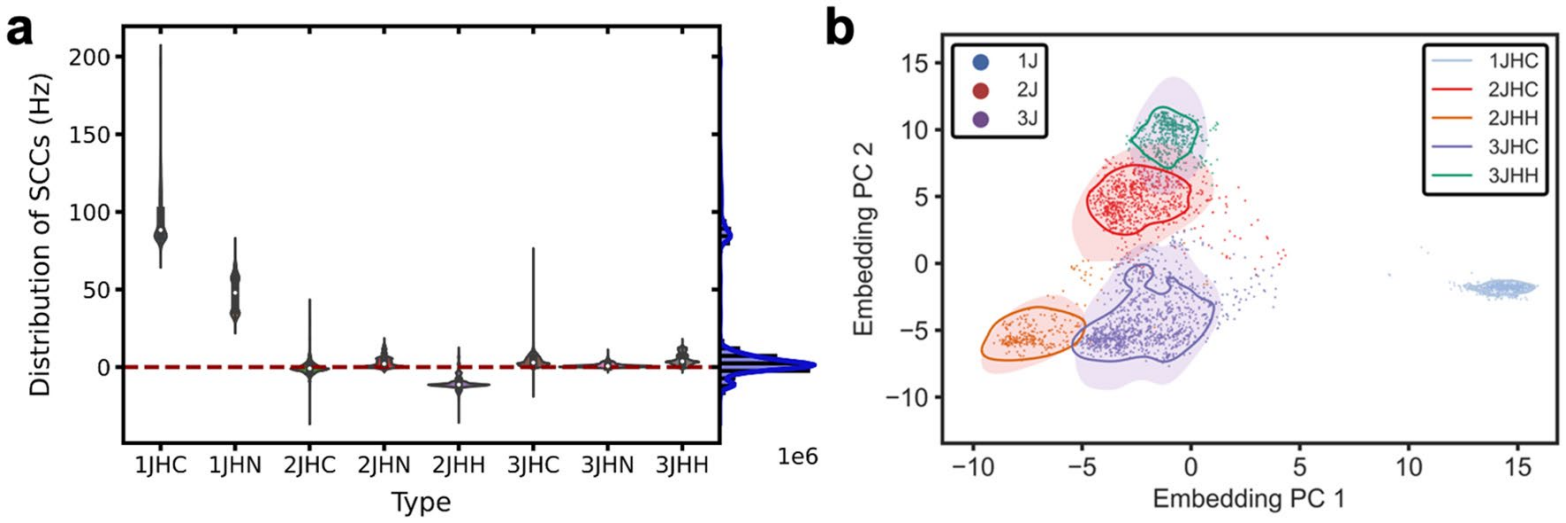
The embedding of GAANN. (**a**) Distribution of 8 types of coupling constants in the violin plot. The kernel density distribution of SCCs is shown on the right. Most of values gather around 0 Hz. The coupling type value of 1 J is significantly higher than that of 2 J and 3 J. The coupling constant values of 2 J and 3 J are concentrated near 0 value, and the difference of them is subtle. (**b**) Embedding learning of GAANN. SCCs are represented as individual points in the two-dimension plane. Shaded areas are the distribution of kernel density estimates of 1 J, 2 J and 3 J coupling, and there is not much overlap between 2 and 3 J coupling. Enclosed contoured lines are the distribution of kernel density estimates of ^1^J_CH_, ^2^J_CH_, ^2^J_HH_, ^3^J_CH_, ^3^J_HH_. The regions surrounded by the five coupling kernel density contours, separate each other clearly.

In order to further explore the predictive performance of GAANN, we analyze the representation learning of GAANN. GAANN's prediction results depend on the quality of its representation learning, which is often referred to as embedded learning^38,39^. Above all, we demonstrate the distribution of SCC target data. As shown in the violin plot of Fig. 4a, the distribution of 8 types of coupling constants is compared based on predicting molecular properties dataset^14^. Most of values gather around 0 Hz according to density distribution of SCCs. On the whole, the value of 1 J is significantly higher than that of 2 J and 3 J. This result indicates that the coupling interaction is stronger with fewer number of the coupling bonds generally. The coupling constant values of 2 J and 3 J are concentrated near 0 value, and the difference of them is subtle.

To qualitatively visualize embedding learning of SCCs, we use principal component analysis^40^ (PCA) to reduce its dimension into two-dimension. As shown in Fig. 4b, the GAANN neural network's embedding learning gives insights that the network has a strong learning ability. Shaded areas are the distribution of kernel density estimates of 1 J, 2 J, and 3 J coupling. Contoured areas are the distribution of kernel density estimates of ^1^J_CH_, ^2^J_CH_, ^2^J_HH_, ^3^J_CH_, ^3^J_HH_. As can be seen from the embedding shadow position, 1 J is much higher than 2 J and 3 J on the whole, therefore the embedding position of 1 J locates on the right side of the diagram alone. Besides, there is not much overlap between 2 J coupling and 3 J coupling though the difference of 2 J and 3 J is very subtle. Moreover, the regions, surrounded by the five coupling kernel density contours, separate each other clearly. In conclusion, GAANN exhibits a strong learning ability to identify the various coupling types and can predict SCCs accurately.

### The interpretability of angles

According to the performance and embedding learning of GAANN, we find that GAANN has a strong predictive performance. Compared with the general GNN models, we introduce relevant angle factors into GAANN which can simulate unique three-dimensional molecular structure better. To demonstrate the interpretability of angles, we carry out ablation experiments on the mechanism of bond angle attention and angle features to testify the importance of relevant angles. Due to the lack of scalar coupling constant values in the test dataset, we chose the train dataset as the whole data to carry out the ablation experiment. In the Fig. 4a, four contrasting loss curves show the prediction error drops from −2.34 to −2.43 by adding bond angle attention mechanism and angle features. The lower loss means the higher accuracy. The green curve is the benchmark which neither adding bond angle attention nor adding angle features. The blue curve and the yellow curve are the models adding only bond angle attention and angle features respectively. The red curve corresponds to the complete GAANN which achieved the highest accuracy among the four models. Notwithstanding little improvement of accuracy, comparative results verify the effect of relevant angles.

**Figure 5:**
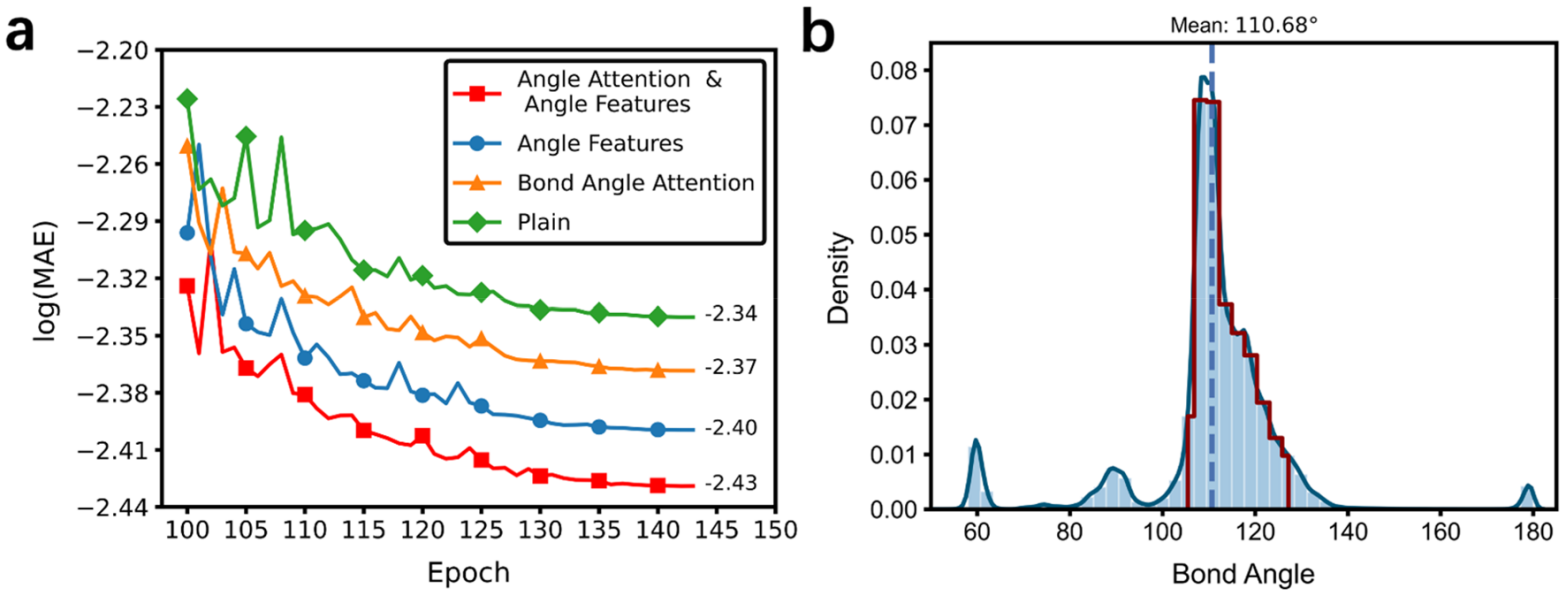
Four contrastive loss curves about angles and statistical distribution of bond angles. (**a**) Loss curves of four contrastive experiments. The lower loss means the higher accuracy. The red loss curves show the best of prediction performances among four contrastive experiments. (**b**) The statistical distribution of bond angles about small molecules with up to 30 carbon atoms. The bond angles concentrate between 103° and 126° in the red line.

In the Fig. 5a, we find that only adding bond angle attention also reduces the prediction error from −2.43 to −2.40. Based on predicting molecular properties dataset^14^, we analyze the distribution of bond angles as shown in Fig. 5b.The bond angles concentrate between 103° and 126° in the red line. We infer the rationale of bond angle attention mechanism that angle attention mechanism assigned different relevance scores of the surrounding atoms to distinguish the subtle differences between bond angles and can reflect the molecule's unique topological structure accurately. Therefore, the bond angle attentional mechanism, added to the GAANN model as a priori physicochemical knowledge, allows GAANN to sufficiently simulate the molecular structure.

In addition, we also find that only adding angle features reduces the prediction error from −2.43 to −2.37 in the Fig. 5a. Although adding angle features and bond angle attention are both inspired by the Karplus equation, the former's performance is better than that of the latter. We infer that angle features include dihedral angles, bond angles and geometric angles, whereas bond angle attention just focuses on the bond angles.

From analysis of the comparative data, we know that the angle features play a crucial role, but it is not clear which of angle features play the main role among the angle features. Therefore, we need to make a data analysis of the relevant angle features (dihedral angle, bond angle, geometric angles).

### Angle feature analysis

**Figure 6:**
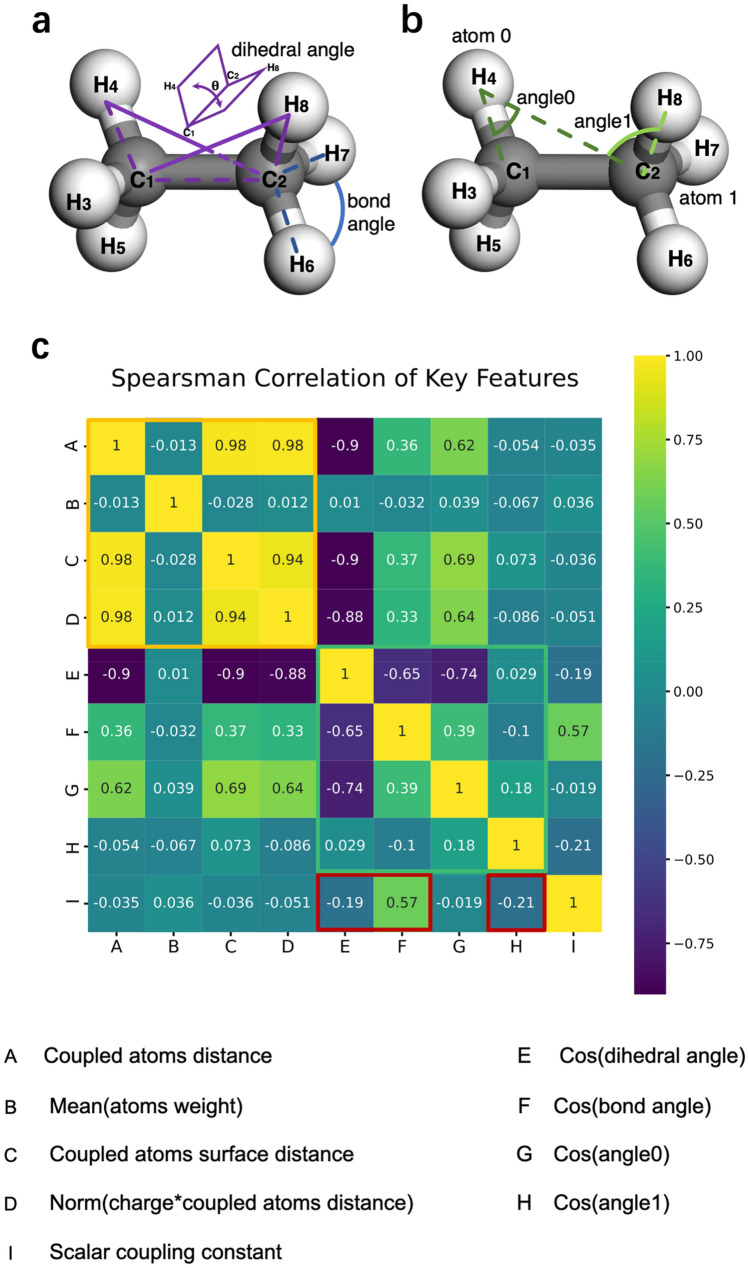
The description of relevant angles and Spearman correlation of key coupling features. (**a**) The description of dihedral angle and bond angle. The purple line describes the dihedral angle between the plane (H_4_-C_1_-C_2_)- and the plane (H_8_-C_2_-C_1_) formed by the 3 J coupled atoms (H_4_ and H_8_). The blue line describes the bond angles ($\angle$ H_6_C_2_H_7_) formed by the 2 J coupled atoms (H_6_ and H_7_). (**b**) The description of the geometric angles. The green line describes angle0 and angle1, which are the geometric angles formed by coupled atoms (H_4_ and C_2_) and their nearest atom of atom0 (H_4_) or atom1 (C_2_). (**c**) Spearman correlation of key coupling features. In the last line, the importance of the relevant angle features to SCC are prominent. Remarkably, the cos(bond angle) has the highest correlation with the SCC among all angle features.

In order to explore which of angle features play the main role among all angle features. Above all, we illustrate the descriptions of all relevant angle. The dihedral angle and bond angle are shown in Fig. 6a, and geometric angles (angle0 and angle1) are shown in Fig. 6b. Noteworthy, the geometric angles are formed by two coupled atoms and their nearest neighbor atom. The centered atom of angle0 ($\angle$ C_1_H_4_C_2_) is the coupled atom0 (H_4_), and the bonding atoms of angle0 consist of the coupled atom1 (C_2_) and the nearest neighbor atom (C_1_) of atom0. Similarly, angle1 ($\angle$ H_4_C_2_H_8_) is defined as the same as angle0.

Spearman correlation coefficient can be used to evaluate the nonlinear correlation between various features^41,42^. As shown in Fig. 6c, we calculated the Spearman correlation coefficient between the key coupling features and the SCC. A (Coupled atoms distances) is highly correlated with C (Coupled atoms surface distance) and D (Norm(charge*coupled atoms distance)), which is consistent with common sense. Besides, that distance-related features (A, C, D) are negatively correlated with SCC (I) on the last line. This is consistent with the prior physicochemical knowledge that the larger the distance, the smaller the coupling magnetic interaction.

As shown on the last line, when the distance between atoms is within the bonding range, the importance of the distance feature decreases, and the importance of the relevant angle features become prominent. Remarkably, the cos(bond angle) has the highest correlation with the SCC. Therefore, we asume that the bond angle is the most important factor for predicting SCCs among all relevant angle features.

### Bond angle for SCC

In addition, the bond angle attention mechanism in GAANN also proves the importance of bond angles. Attention mechanism is a method to train the model to pay attention to the coupled atoms’ local specific structural environment^35^. Bond angle attention, based on the prior knowledge of bond angle, is designed to simulate the various and specific bond structure of molecules.

**Figure 7:**
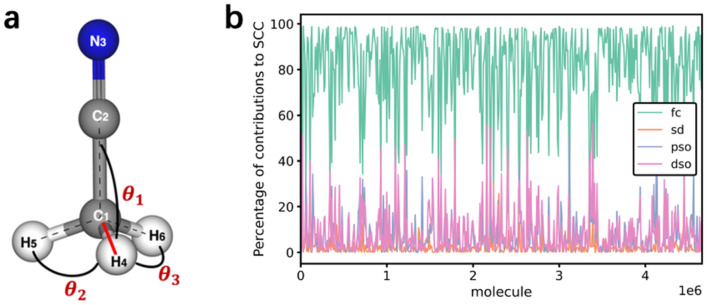
Bond angle for SCC. (**a**) Bond angle attention mechanism. $\theta_{i} (\theta_{1} ,\theta_{2} \;{\text{and}}\;\theta_{3} )$ represent the bond angles between the bond $(\overrightarrow {{H_{4} C_{1} }} )$ and the adjacent bonded atoms (C_2_, H_5_ and H_6_) respectively. (**b**) Percentage of contributions to SCC. SCC is in most cases determined by the Fermi Contact.

The detail calculation of bond angle attention mechanism is shown in Fig. 7a. When we want to simulate the unique effect of the directed bond $(\overrightarrow {{H_{4} C_{1} }} )$ on the central atom (C_1_), we need to set the weight coefficient *attn* for the directed bond $(\overrightarrow {{H_{4} C_{1} }} )$. The coefficient *attn* reflects the comprehensive contribution of neighboring bonds to the central atom. However, the comprehensive contributions are affected by many factors, it is difficult to obtain a universal law like Karplus equation or criterion to generalize this effect. Excitingly, the Karplus equation kindles another enlightenment. The Karplus equation 3$J = A*\cos^{2} \varphi + B*\cos \varphi + C,$ which bridges dihedral angles and the corresponding 3 J coupling constants. Thereinto, the values of A, B, and C reflect the comprehensive effect of various factors (such as hybrid orbitals, electronegativity of atoms) in molecules.

Although we cannot accurately calculate the values of A, B, and C as the Karplus equation does, we can generalize the comprehensive effects of various factors in an AI data-driven way. Through data-driven, AI automatically learns to obtain potential complex influencing factors and summarizes the specific influencing results. When we can't figure out the complex effects within the system, the idea of generalizing a scientific rule in an AI data-driven way is worth popularizing. Therefore, we set the value of *attn* as the contribution weight of each bond. The specific calculation of *attn* is shown in the Formula 1. The cosine of angles can usually be understood as an irrelevant relation between the influencing factors and the angles. $\theta_{i} (\theta_{1} ,\theta_{2} \;{\text{and}}\;\theta_{3} )$ represent the bond angles between the bond $(\overrightarrow {{H_{4} C_{1} }} )$ and the adjacent bonded atoms (C_2_, H_5_ and H_6_) respectively. *N*(*e*_*vw*_) denotes the set of neighboring bonds of the centered bond $(\overrightarrow {{H_{4} C_{1} }} )$. M denotes the number of neighboring bond angles.

1 $$attn = \mathop \sum \limits_{{i \in N\left( {e_{wv} } \right)}} \frac{{A^{\prime}cos^{2} {\uptheta }_{i} + B^{\prime}cos{\uptheta }_{i} + C^{\prime}}}{M}$$

In the Formula 1, the above coefficients *A*′, *B*′, and *C*′ are self-learned, and $A^{\prime } \approx 0.12,B^{\prime } \approx 0.99,C^{\prime } \approx 0.27$ are learned from the GAANN. From the magnitude of the *A*′, *B*′, and *C*′ coefficients, it is the first cosine of the bond angle that matters most, not the second cosine. Meanwhile, it also can be concluded that bond angle does play an important role in predicting SCC according to the coefficient of *B*′. This result can further support the result of Spearman's coefficient mentioned above.

Quantum mechanically, the SCC is the sum of the Fermi contact interaction (fc), the spin–dipolar interaction (sd), the paramagnetic spin–orbit interaction (pso) and the diamagnetic spin–orbit interaction (dso)^10^. We analyzed the percentages of the four terms to SCC in the dataset. As is shown in Fig. 7b, the SCC is in most cases determined by the Fermi contact interaction which is an interaction between an electron and an atomic nucleus^10^. As mentioned above, the bond angle plays an importance role of predicting SCC. We infer that the bond angle is closely related to the Fermi contact contribution. From the perspective of molecular structure, the magnitude of the bond angle can be considered as a manifestation of the equilibrium of multiple bond systems.

## Discussion

In the paper, we propose the GAANN model to predict SCCs, and GAANN achieves a high prediction accuracy of near DFT calculation under the condition of log(MAE) of −2.52. Moreover, GAANN focus on the physical interpretability of angles and demonstrates that bond angle has the highest correlation with the SCC among all relevant angle features of three applicable coupling types. From the perspective of AI, this result is consistent with the covalent bond theory.

We now discussion several potential limitations of the methods and possible ways to overcome them. First, a good result cannot be separated from a good model, but also from good feature data. We carry out feature engineering with the guidance of prior physicochemical knowledge. Inspired by Karplus equation, we obtain an enlightenment that relevant angles play a crucial role of influencing all types of SCCs (1 J, 2 J, 3 J and etc.). Besides, angle-related features offer unique three-dimension molecular structural information which are important to predict the SCC. In this way, feature engineering is carried out as far as possible according to existing prior knowledge. It is worth noting that we mainly wanted to explore the influence of molecular structure information on the SCC. Therefore, we mainly focused on the features related to molecular structure in the feature engineering, ignoring the electronic or magnetic features such as Mulliken charge and magnetic shielding tensor. We believe that the structural characteristics of molecules are the result of the integrated manifestation of all the influencing factors. The influence of these factors is included in the structural information.

Second, since SCCs are affected by many factors, it is difficult to obtain a universal law like Karplus equation. However, we can explore some key factors that affect the SCC, and then build a heuristic AI model to predict the SCC. When we can't figure out the complex effects within the system, the idea of generalizing a scientific rule in an AI data-driven way is worth popularizing. In order to further improve the prediction performance of GAANN, we are trying to design more concise AI model that combines with enough prior physicochemical knowledge to achieve satisfactory accuracy. In this way, we can avoid falling into a dilemma, where ignoring scientific theorems makes the AI model runs counter to the accumulated wisdom of mankind.

## Methods

The GAANN model has been trained by Fastai library^43^ in PyTorch framework. The complete model was trained for 144 epochs using the one-cycle learning rate policy. We optimized performance by tuning parameters toward higher accuracy without over-fitting (see Supplementary Table 2 for more model parameters). All the molecular stereo structures in this paper were drawn by the “Vesta” software (OpenGL version: 2.1 ATI-4.5.14, http://www.jp-minerals.org/vesta/en/download.html).

### Data source

The dataset we used to predict SCCs is provided by Kaggle competition and this dataset is part of the recognized QM9 dataset^14^. The competition web site is https://www.kaggle.com/c/champs-scalar-coupling. The dataset contains 130,775 unique molecules, all of which contain only five types of atoms: carbon (C), hydrogen (H), nitrogen (N), fluorine (F), and oxygen (O). And there are eight different coupling types (^1^J_CH_, ^1^J_NH_, ^2^J_CH_, ^2^J_NH_, ^2^J_HH_, ^3^J_CH_, ^3^J_NH_, ^3^J_HH_) in the dataset. In the competition, the train dataset contains 85,003 unique molecules with 4,658,147 scalar coupling values. Each molecule contains a wide variety of SCCs. The test dataset contains 45,772 molecules whose scalar coupling values were lacking. These lacking 2,505,542 values need to be predicted then submit to the competition organizer to evaluating predictive performance. In order to test whether the GAANN model can predict more complex unknown molecules, the molecules in the test dataset are more complex than those in the train dataset. The dataset mainly includes molecule structure files (XYZ files) and corresponding goal SCCs. Molecule structure files include cartesian coordinates and meta-information of every atom^14^.

### Features engineering

There are much of relevant information that affect the SCCs. We can extract relevant feature information (such as atom type, bond type, distance, atomic number, hybridization and etc.) in XYZ files with Python packages RDKit and DeepChem library^44^. All features which potentially influence SCCs are extracted and engineered with the guidance of prior physicochemical knowledge^9,13,36^. Inspired by Karplus Equation, angle-related features, offering unique three-dimension molecular structural information, are indispensable apart from the interatomic distance features and the atom's intrinsic property features. We divide these factors into four features categories: atom features, bond features, coupling edge features and molecular features (see Supplementary Table 1, all features with their descriptions and sizes are listed).

### Message passing structure

As part of the encoder, MPNN^31^ is applied to transfer and integrate molecular graph features. The clever thing of GAANN is that two graph message passing layers are used to handle two graphical inputs, as shown in Fig. 2. One is a molecular bond graph, and the other is a coupling graph, where nodes represent atoms and edges represent scalar coupling atom pairs.

In graph representation of MPNN, *v* refers to atom vertices and *e* to edges. The initial atom and edge state features see Supplementary Table 1, and their initial feature vectors’ sizes are extended to *d*_*model*_ and $d_{model} \times \frac{{d_{model} }}{2}$ by linear transformation before the message passing layer. Edges can be the real chemical bonds or virtual scalar coupling edges. In bond message passing layer, edges are the chemical bonds. Atoms’ hidden states $h_{v}^{t} \in R^{{d_{model} }}$ are iteratively updated by edges’ hidden states $e_{vw} \in R^{{d_{{model \times \frac{{d_{model} }}{2}}} }}$, as shown in formula 3. Herein, *w* is one of *v*’s neighbor atoms.

2 $$h_{v}^{t + 1} = \mathop \sum \limits_{w \in N\left( v \right)} attn{*}Conv\left( {h_{w}^{t} } \right){*}e_{vw} + h_{v}^{t}$$

3 $$h_{v}^{t + 1} = \mathop \sum \limits_{w \in N\left( v \right)} Conv\left( {h_{w}^{t} } \right){*}e_{vw} + h_{v}^{t}$$

Here *N*(*v*) is the set of neighboring atoms of *v* and *t* is the number of message passing layer. *Conv* is the convolution function that including padding with zero values and unfolding operations to reshape the atom feature vectors to match with the size of edge features matrix *e*_*vw*_. Finally, $Conv \left( {h_{v}^{t} } \right){*} e_{vw} \in R^{{d_{{model \times \frac{{d_{model} }}{2}}} }}$ is conducted with angle attention coefficient *attn*. The state information of each atom is influenced by the bonds around it.

After the bond message passing layer, a similar mechanism was applied to update these hidden scalar coupling edge states in scalar coupling message passing layer, but the difference is that this process does not use an angle attention mechanism, as shown in formula 3. The reason is that the bond angles are primarily worked by chemical bond. After *T* times of bond and scalar coupling message passing iteratively, the final coupled atomic features $h_{v}^{T}$ contain the atomic property information and enough local environment information.

### Self-attention structure

Self-attention mechanism is a special attention mechanism, which is different from standard attention mechanism. Self-attention mechanism introduced scaled dot-product attention and multi-head attention, and it can flexibly handle sequences of different lengths. Hence it is perfect for dealing with molecular diversity of various molecular structures in the GAANN, as shown in Fig. 2.

In the scaled dot-product attention, each of the input vector $h_{v}^{T} \in R^{{d_{model} }}$ creates Query (*Q*), Key (*K*) and Value (*V*) three vectors respectively, which are linearly transformed by three different parameters. The output of attention is a weighted average of the input values, where the weight is determined by the similarity of the query vector and key’s transpose vectors. Firstly, the dot product of $Q \in R^{{d_{model} \times 1}}$ and $K^{T} \in R^{{1 \times d_{model} }}$ is calculated, and then the dot product is scaled. *d*_*model*_ is the dimension of the input vector. In the dot product operation, scaling can prevent the dot product from being too large. Then, the scaled dot product is normalized into a probability distribution by *softmax* function. Finally, the attention score is obtained by multiplying the normalized dot product with matrix $V \in R^{{d_{model} }}$. The output $O_{i} \in R^{{d_{model} }}$ of the scaled dot product attention layer can be expressed by the formula 4:

4 $$Attention\left( {Q, K, V} \right) = O_{i} = softmax\left( {\frac{{QK^{T} }}{{\sqrt {d_{model} } }}} \right)V$$

The self-attention layer is further improved by using the multi-head mechanism. Before scaled dot-product attention, the input eigenvectors are projected onto different subspaces through linear transformation to make every head have different emphasis on all the information, it will make information network to extract more comprehensive. In the multi-head mechanism, each head structure of self-attention is the same and can be calculated in parallel, but it has different weights. After parallel calculation, multi-head attention concatenates the *h* attention results $O_{i} \in R^{{ \frac{{d_{model} }}{h} \times 1}}$. Multi-head self-attention is shown in the formula 5, where $W \in R^{{d_{model} \times d_{model} }}$ is the linear transformation parameter of the multilayer perceptron.

5 $$f_{atom} = Multihead\left( {Q, K, V } \right) = Concat\left( {O_{1} , O_{2} , \ldots , O_{h} } \right)W$$

The purpose of self-attention^27^ mechanism is to select more critical information from the global information, so it can make good use of global characteristic information instead of local characteristic information. Comparing with traditional attention mechanism, self-attention mechanism can capture relationships between all atoms automatically, no matter how far apart the two atoms are. For this reason, self-attention mechanism can deal with the long distance and multi-level dependency relationship in the complex macromolecular structures.

Subsequently, to denote the interaction relevantly, each virtual coupled edge is concatenated by two coupled atoms and whole molecular features (see Supplementary Table 1 for all features) before the decoder part. Herein, $f_{atom} \in R^{{d_{model} \times 1}} ,f_{mol} \in R^{{ \frac{{d_{model} }}{2} \times 1}} .$ In formula 6, a MLP is used to predict the SCC in the decoding process. Predicting SCC is a regression problem and log(MAE) is selected as evaluation criteria of prediction performance.

6 $$SCC = MLP\left( {f_{atom1} \oplus f_{atom2} \oplus f_{mol} } \right)$$

## Supplementary Information

Supplementary Information.

## Data Availability

The data that support the findings of this study are available in https://www.kaggle.com/c/champs-scalar-coupling/data.

## Code availability

The source code of our proposed GAANN model is available at https://github.com/FangJia0901/Bond-Angle-for-SCCs.
