# Supplementary material for: Predicting scalar coupling constants by graph angle-attention neural network
Source: Sci Rep. 2021 Sep 21;11:18686. doi: 10.1038/s41598-021-97146-1 (PMC8455698; doi:10.1038/s41598-021-97146-1)
Supplement: Supplementary file 1 — Supplementary Information. [file 41598_2021_97146_MOESM1_ESM.docx]

Here, we enumerate the relevant features as well as the corresponding descriptions, coding styles, and coding sizes in Supplementary Table 1. We extract those features information with Python packages RDKit and DeepChem library based on the molecule structure files (XYZ files). To distinguish clearly, we divide those features into four categories: atom features, bond features, coupling edge features, and molecular features. Therein, molecular features include coupling edge features. Furthermore, because those features are input step by step in GAANN, we categorize those features in sequence, instead of inputting all features at one time just like the general machine learning model.

Supplementary Table 1: All Features of each coupled atomic pair

| **Atom Features** | | **Description** | **Size** |
| --- | --- | --- | --- |
| Atom type | | H, C, O, F, N (one-hot) | 5 |
| Degree | | 1,2,3,4,5 (one-hot) | 5 |
| Hybridization | | SP, SP^2^, SP^3^, null (one-hot) | 4 |
| InAromatic | | Whether the atom is in an aromatic system (one-hot) | 1 |
| Formal charge | | The charge equally assigned to an atom in a molecule (number) | 1 |
| Weight | | Atomic weight (integer) | 1 |
| Mean(neighbor atomic weight) | | The average atomic weight of adjacent atoms (number) | 1 |
| Mean(neighbor bond length) | | The average bond length of adjacent atoms (number) | 1 |
| **Bond Features** | | **Description** | **Size** |
| Bond type | | Single, double, triple (one-hot) | 3 |
| IsAromatic | | Whether the bond is part of an aromatic system (one-hot) | 1 |
| IsConjugated | | Whether the bond is part of conjugation (binary) | 1 |
| IsRing | | Whether the bond is part of ring (binary) | 1 |
| Bond length | | The distance between two covalent bonded atoms (float) | 1 |
| Norm(bond length) | | Normalized bond length of two covalent bonded atoms (float) | 1 |
| **Coupling Edge Features** | | **Description** | **Size** |
| Coupling type | | ^1^J_CH_，^1^J_NH_，^2^J_CH_，^2^J_NH_，^2^J_HH_，^3^J_CH_，^3^J_NH_，^3^J_HH_ (one-hot) | 8 |
| Angle | Cos(dihedral angle) | Cosine of dihedral angle for a 3 J coupling (number) | 1 |
|  | Cos(bond angle) | Cosine of bond angle for a 2 J coupling (number) | 1 |
|  | Cos(angle1) | Cosine of the nearest angle to atom 0 (number) | 1 |
|  | Cos(angle0) | Cosine of the nearest angle to atom 1 (number) | 1 |
| Coupled atoms distance | | Distance between the two coupled atoms (number) | 1 |
| Norm(coupled atoms distance) | | Normalized distance between the two coupled atoms (number) | 1 |
| Coupled atoms surface distance | | Surface distance between two coupled atoms (number) | 1 |
| Charge*coupled atoms distance | | The product of electric charge and coupled atoms distance (number) | 1 |
| Norm(coupled atoms distance)  *mass | | The product of mass of coupled atom pair and normalized distance between two coupled atoms (float) | 1 |
| **Molecules Features** | | **Description** | **Size** |
| Coupling edge features | | Scalar coupling edge features | 16 |
| Atom number | | Total number of atoms in a molecule (integer) | 1 |
| X atom number | | The number of X (X = C, F, H, N, O) (integer) | 5 |
| Std(mol bond length) | | The standard deviation of all bond lengths in a molecule (float) | 1 |
| Mean(mol bond length) | | The average of all bond lengths in a molecule (float) | 1 |
| Mean(mol atom weight) | | The average of all atom weights in a molecule (float) | 1 |

We enumerate the hyperparameters applied on the GAANN model in Supplementary Table 2 and explain the parameters' meanings and values. This hyperparameters is the best result after many experiments under the condition that GAANN obtains high prediction accuracy without overfit or underfit.

Supplementary Table 2: Hyper-parameters of GAANN model

| Hyperparameters | Descriptors | Default Value |
| --- | --- | --- |
| T | The number of stacked layers of the encoding structure | 6 |
| $d_{model}$ | The dimension of the model during passing | 512 |
| Epoch | Number of iterations of the overall training data | 144 |
| Batch size | The number of samples selected for an iteration training | 64 |
| Learning rate | The speed of a network updating its parameters | 0.002 |
| Attention heads | Number of channels in self-attention | 4 |
| Weight decay | A regularization term that penalizes big weights | 1e-5 |
| Dropout | Regularization technique to avoid over-fitting | 0.001 |
